# Supplementary material for: Global patterns of rebound to normal RSV dynamics following COVID-19 suppression
Source: BMC Infect Dis. 2024 Jun 25;24:635. doi: 10.1186/s12879-024-09509-4 (PMC11201371; doi:10.1186/s12879-024-09509-4)
Supplement: Supplementary file 1 — Supplementary Material 1 [file 12879_2024_9509_MOESM1_ESM.docx]

**Title**: Global patterns of rebound to normal RSV dynamics following COVID-19 suppression

Supplementary to Thindwa et. al.

**Correspondence**

*[deus.thindwa@yale.edu](mailto:deus.thindwa@yale.edu)

*[daniel.weinberger@yale.edu](mailto:daniel.weinberger@yale.edu)

## Supplementary Text 1: Generalised Additive Model

**Generalised additive modelling with P-spline**

Given the time series data from 28 countries that met the criteria of reporting at least 100 annual RSV cases between 2017 and 2023, we fitted generalized additive models with penalised B-spline (P-spline). This threshold was chosen empirically because models did not consistently converge when fit to data from countries with more sparse data. Model fitting was facilitated by the “mgcv” and “pspline.inference” R packages [9–11]. In brief, splines were employed to capture the unknown non-linear shapes/functions of the time series due to COVID-19 disruption of the normal RSV seasonal patterns. Penalised B-splines (P-splines) were used to avoid under- and over-fitting through use of discrete penalties on the basis coefficients [12]. We fitted a log-linked GAM with a Poisson likelihood using maximum likelihood based on the framework that was developed for outbreak time series (pspline.inference) [10], which simulates a specified number of samples from the GAM model to obtain the median outcome and its uncertainty. We selected as many knots as possible (=25 knots) on a grid of equally spaced quantiles. A penalty parameter, which controls smoothing and therefore the minimum required number of knots for an optimal fit to the data, was selected automatically via random effect maximum likelihood (REML). Sensitivity analysis on the number of knots selection for the P-spline is given in (Figure S13).

Weekly RSV cases were assumed to follow a Poisson distribution with mean ($\mu$) as follows.

$y_{i} \sim Poisson\left( \mu_{i} \right)$,

where $\mu$ is equal to the expectation of $y_{i}$:

𝜇 = $\mathbb{E(}y_{i}$), and

$\mathrm{Log}\left( \mu\right) = \beta_{0} + f\left( t \right)$.

$Log(\mu)$ is equal to the intercept ($\beta_{0}$) plus the smooth function of weekly cases ($f\left( t \right))$ estimated using the P-splines.

## Supplementary Text 2: RSV dynamic time warping and classification

**Dynamic Time Warping**

We adopted a shape-based time series clustering approach using dynamic time warping (DTW) to quantify dissimilarity between any two normalised fitted P-spline time-series (the query-*Q* and reference-*R*) from 26 countries, globally [1]. The DTW algorithm computed the optimum warping path (minimum distance) between two time-series under certain constraints including monotonicity, continuity, warping window and boundary. The algorithm initially aligned the two time-series sequences based on common features before computing distances (Figure 1B). The *dtwclust* and *dtw* R packages facilitated implementation of the algorithm and optimisation [2, 3].

**Local Cost Matrix (LCM)**

After computing distances, a Local Cost Matrix (LCM) was created with $n$ × $m$ dimensions, corresponding to the length of the time series, for every pair of time-series compared. Given the $Q$ and $R$ input time-series, for each element $(i,j)$ of the *LCM,* as shown in (Figure 1B), the distance $l_{p}$ norm (where $p=2$ for Euclidean as opposed to $p=1$ for Manhattan distances) between $Q_{i}$ and $R_{j}$ was computed as

$$LCM\left( i,j \right)=\left( \sum{|Q_{i}-R_{j}|}^{p} \right)^{1/p}$$

Hence, the DTW algorithm identified the path that minimizes the alignment between $Q$ and $R$ by iteratively stepping through the LCM, starting at $LCM\left( 1,1 \right)$ and finishing at $LCM\left( n,m \right)$, and aggregating the cost. At each step, the algorithm found the direction in which the cost increases the least under the above constraints. We defined $\varphi=\{\left( 1,1 \right), \ldots,(n,m)\}$ as a set containing all the points that fell on the optimum path, with the final distance computed as below and facilitated by *proxy* R package [4], where $m_{\varphi}$ is a per-step weighting coefficient and $M_{\varphi}$ is the corresponding normalization constant:

$${DTW}_{p}\left( Q,R \right)=\left( \sum\frac{m_{\varphi}{LCM(k)}^{p}}{M_{\varphi}} \right)^{1/p}, \forall k \in\varphi$$

**DTW window size**

To limit the area of the LCM that can be reached by the DTW algorithm to marginally speed up the DTW calculation and match RSV dynamics in nearby seasons, we implemented the Sakoe-Chiba window as a global constraint [5], with which the allowed region was created along the diagonal of the LCM (Figure 1B). For a window size $w$, the valid region of the LCM, the slanted band window, constituted all valid points in the range $[\left( i, j-w \right), \left( i, j+w \right)]$ for all $\left( i, j \right)$ along the LCM diagonal, and at each step, $2w+1$ elements fell within the window. To select an optimal window size and number of clusters for hierarchical clustering of countries, we evaluated clustering using Modified Davies-Bouldin (DB) internal cluster validity index (CVI), iterating across different values of window size from 1 to 100 weeks (and the entire warping window) and of all possible number of clusters from 2 to 25 [3, 6, 7]. For each window size and number of clusters, the DB CVI calculated distances from computed cluster centroids (centroid choice is described below).

**Time series prototype or centroid**

We computed an average series or prototype or centroid to define a time-series that effectively summarizes the most important characteristics of all series in a given cluster. Our choice of prototyping function was the DTW barycentre averaging corresponding to DTW distance measure. The DTW barycentre averaging approach randomly selected one of the series in the data as a centroid, such that on each iteration, the DTW alignment between each series in the cluster and centroid was computed. Warping was performed in DTW, and several time-points from a given time-series mapped to a single time-point in the centroid series; Thus, for each time-point in the centroid, all the corresponding values from all series in a cluster were grouped together according to DTW alignments, and the mean was computed for each centroid point using the values contained in each group. This was iteratively repeated until convergence was assumed (Figure S9).

Hierarchical clustering of countries created a hierarchy of groups in which, as the level in the hierarchy increased, clusters were created by merging clusters from the next lower level, such that an ordered sequence of groupings was obtained [8]. The created hierarchy was visualized as a binary tree using a dendrogram, where the height of each node was proportional to the value of the inter-group dissimilarity between its two daughter nodes (Figure 4, Figure S10).

## Supplementary Text 3: RSV seasonal metrics calculation

**Epidemic analysis**

We defined four metrics to summarize RSV epidemics before and after COVID-19 suppression period in each of the 28 countries, given the P-spline fitted cases $f\left( t \right)$ at each time point $t$ (Figure 1).

1. Onset timing (O) was defined as a week when the rate of change of epidemic growth rate increased the most corresponding to the timing of the maximum of the second derivative in the segment of increasing first derivative for the fitted P-spline with respect to time (week); this is mathematically represented as [13]:

$$O=t:Max\left\{ \frac{d}{dt}\left[ \frac{d\left( f\left( t \right) \right)}{dt}>0 \right] \right\}$$

2. Peak timing (P) was defined as the week of maximum wave cases, which corresponds to the timing of the maximum value of the fitted P-spline curve in each epidemic wave [14], represented as:

$P=t:Max\left\{ f\left( t \right) \right\}$

1. Growth rate (G) was defined as the maximum number of new cases per week corresponding to the values of the maximum derivative of the log-transformed fitted P-spline curve with respect to time (week) [15]. This is mathematically represented as:

$$G=Max\left\{ \frac{d}{dt} \left[ log\left( f\left( t \right) \right)>0 \right] \right\}$$

2. Intensity (I) was defined as the relative magnitude of the epidemic peak, corresponding to the integral of the log fitted P-spline curve with respect to time (week) in the segment of positive growth rate. This is equivalent to the sum of the log of the fitted P-spline at the peak minus the start of the epidemic. This is mathematically represented as:

$$I=\int\left\{ \frac{d}{dt} \left[ log\left( f\left( t \right) \right)>0 \right]>0 \right\}dt$$

**Correlation coefficients**

We performed correlation tests between two distinct phases of time series in 28 countries globally to establish the degree of return to normal RSV patterns post COVID-19 suppression. We compared pre-COVID-19 mean onset and peak timings, growth rate and intensity to the first wave, second wave and third wave of RSV epidemic following COVID-19 suppression. Onset and peak timings for pre- and post-COVID-19 phases were quantified using circular correlation coefficients ($c$), as implemented in the “circular” R package [16], and visualised using X-Y plots. For a sample of $n$ pairs of time-series points or angles {($a_{11},a_{21}$), ($a_{12},a_{22}$), …, ($a_{1n},a_{2n}$)} corresponding to the two phases of time-series, the circular correlation is given by:

$$c=\frac{\sum_{k=1}^{n} \sin\left( a_{1k}-T_{11} \right)sin(a_{2k}-T_{21})}{\sqrt{\sum_{k=1}^{n} {sin}^{2} \left( a_{1k}-T_{11} \right)\sum_{k=1}^{n} {sin}^{2} \left( a_{2k}-T_{21} \right)}}$$

where $T_{11}$ is mean direction of the first circular variable and $T_{21}$ is the mean direction of the second circular variable. Uncertainty around circular correlation coefficient was based bootstrapping the estimated correlation coefficient (1000 replicates) with replacement.

Growth rate and intensity for pre- and post-COVID-19 phases were quantified using Pearson’s correlation coefficient ($r$), as implemented in the “stats” R package, and visualised using X-Y plots. For a sample of $n$ pairs of time-series points {($x_{1},y_{1}$), ($x_{2},y_{2}$), …, ($y_{n},y_{n}$)} corresponding to the two phases of time-series, the Pearson’s correlation is given by:

$$r=\frac{\sum_{i=1}^{n} \left( x_{i}-\bar{x} \right) (y_{i}-\bar{y})}{\sqrt{\sum_{i=1}^{n} \left( x_{i}-\bar{x} \right)^{2}\sum_{i=1}^{n} {(y_{i}-\bar{y})}^{2}}}$$

where $\bar{x}=1/n\sum_{i=1}^{n} x_{i}$ is the sample mean, and analogously for $\bar{y}$. Uncertainty around Pearson correlation coefficient was based on Fisher’s transformation method [17].

**Regression models of RSV onset, peak timing, growth rate, and intensity**

Cox Proportional Hazards regression models were separately fitted to ‘time to RSV onset’ and ‘time to RSV peak’ to identify factors associated with the timing of RSV waves, whereas linear models were fitted to ‘RSV growth rate’ and ‘RSV intensity’. Mathematical descriptions of the fully saturated models are given below:

where the baseline hazard represents the hazard when all predictors are equal to 0, and $b_{1},b_{2},\ldots,b_{n}$ are predictor coefficients. Predictors are included or excluded in the multivariate model based on stepwise selection [18]. The hazard ratio (HR) is the ratio of expected hazards between the two comparison groups.

where the intercept represents the expected growth rate or intensity when all predictors are equal to 0, and $b_{1},b_{2},\ldots,b_{n}$ are predictor coefficients. Predictors are included or excluded in the multivariate model based on stepwise selection [18]. The effect size is the ratio of expected values between the two comparison groups.

| 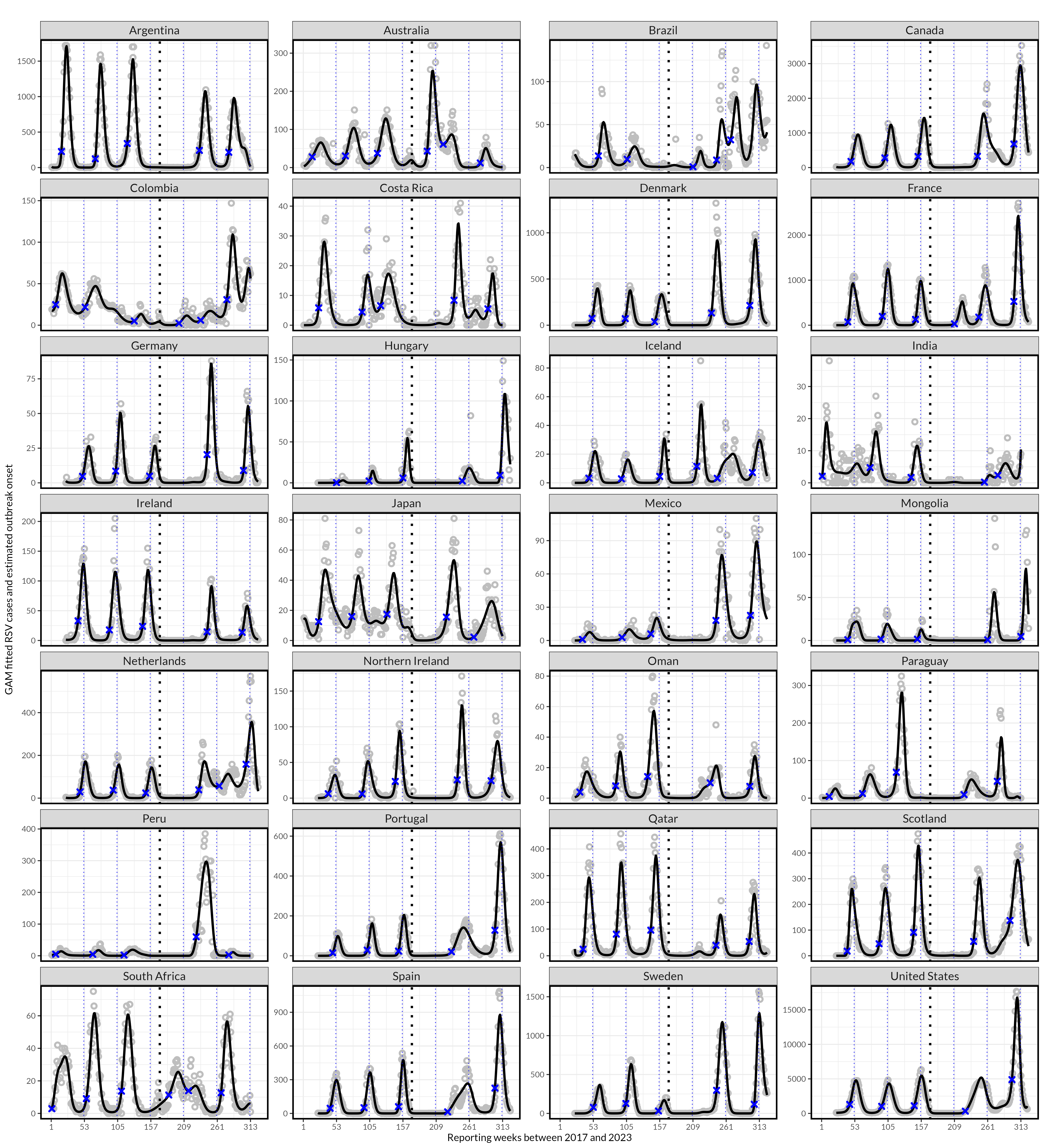 |
| --- |

Supplementary Figure 1. Onset of respiratory syncytial virus (RSV) epidemics across 28 member countries of the World Health Organisation (WHO). The solid black line is the P-spline fitted to observed data represented by gray circles. The dotted black line corresponds to April 2020 at the beginning of COVID-19 pandemic, and the blue star corresponds to the maximum second derivative value in the segment of increasing first derivative of the fitted P-spline GAM, which defines the start of the RSV epidemic (onset) as described in Figure 1.

| 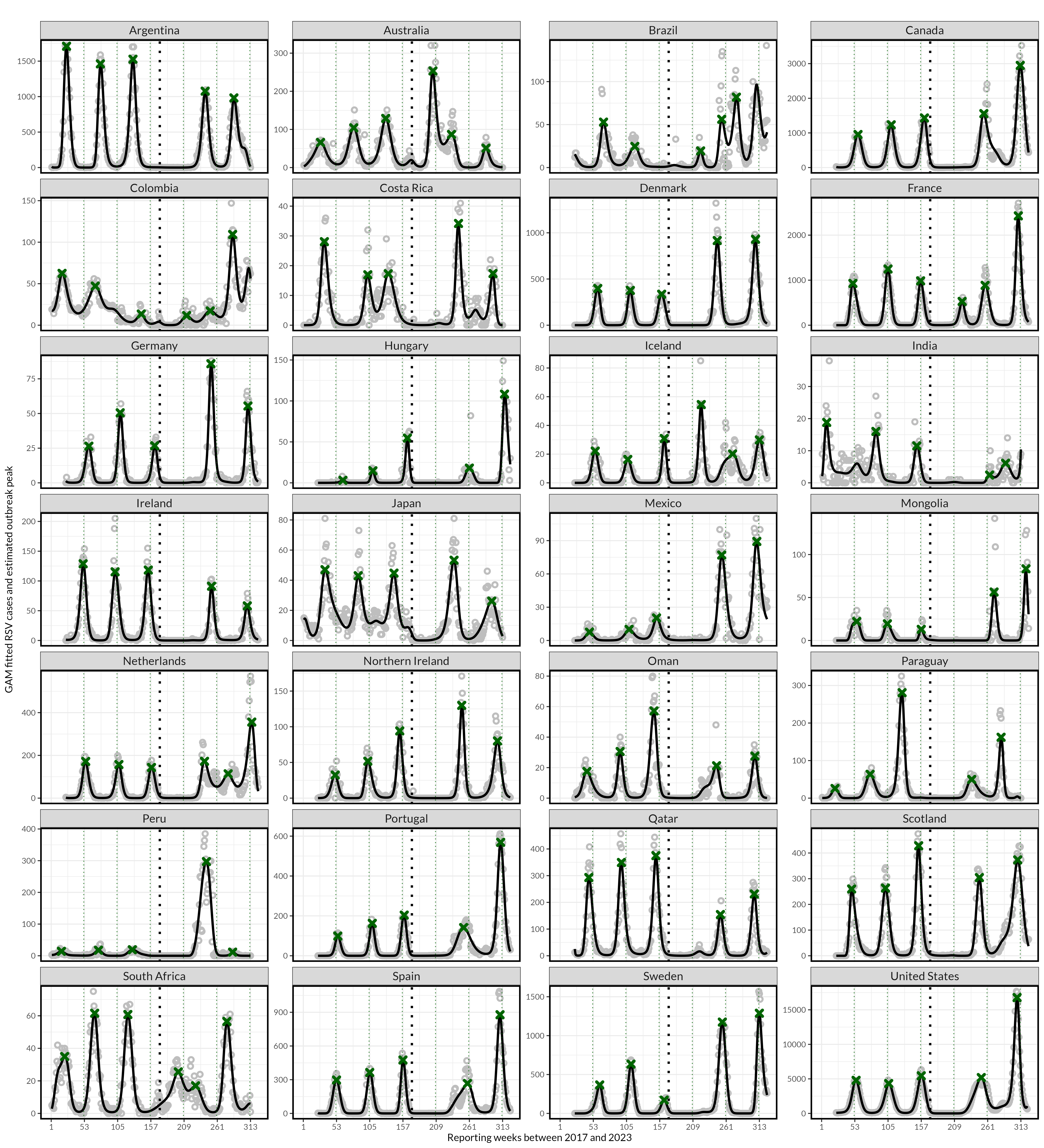 |
| --- |

Supplementary Figure 2. Peak timing of respiratory syncytial virus (RSV) epidemics across 28 member countries of the World Health Organisation (WHO). The solid black line is the P-spline fitted to observed data represented by gray circles. The dotted black line corresponds to April 2020 at the beginning of COVID-19 pandemic, and the green star corresponds to the maximum value of the black fitted P-spline GAM, defining the wave peak of RSV cases.

| 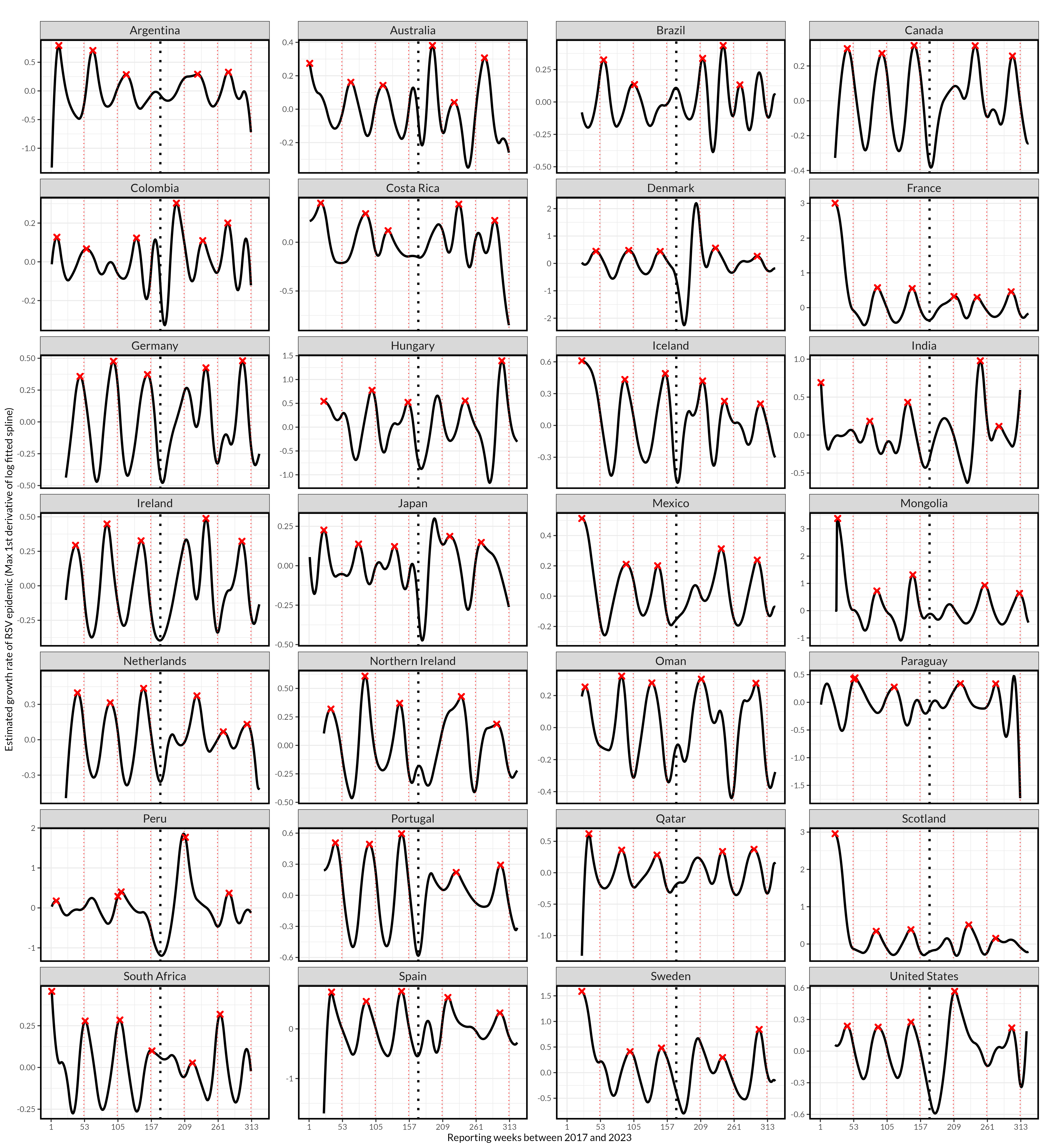 |
| --- |

Supplementary Figure 3. Growth rate of respiratory syncytial virus (RSV) epidemics across 28 member countries of the World Health Organisation (WHO). The solid black line corresponds to the first derivative of the log of the fitted P-spline GAM, the dotted black line corresponds to April 2020 at the beginning of COVID-19 pandemic, and the red star corresponds to the maximum value of the black line which defines the maximum number of new cases per week (growth rate) as described in Figure 1.

| 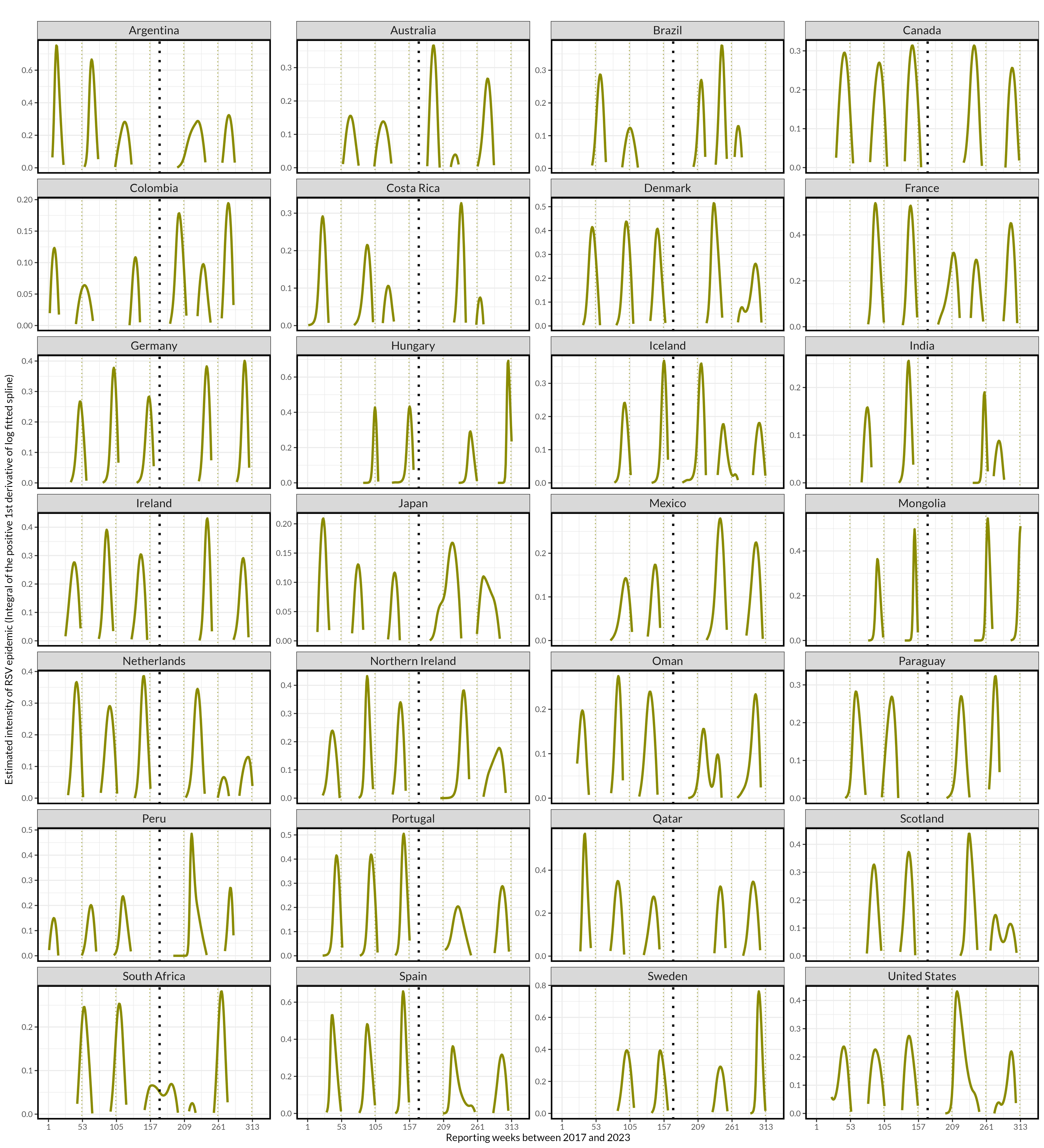 |
| --- |

Supplementary Figure 4. Intensity of respiratory syncytial virus (RSV) epidemics across 28 member countries of the World Health Organisation (WHO). The dotted black line corresponds to April 2020 at the beginning of COVID-19 pandemic, and the area under the curve indicates the relative magnitude of RSV cases in each wave corresponding to the integral of the positive derivative of the log fitted black GAM P-spline before epidemic peak timing.

| 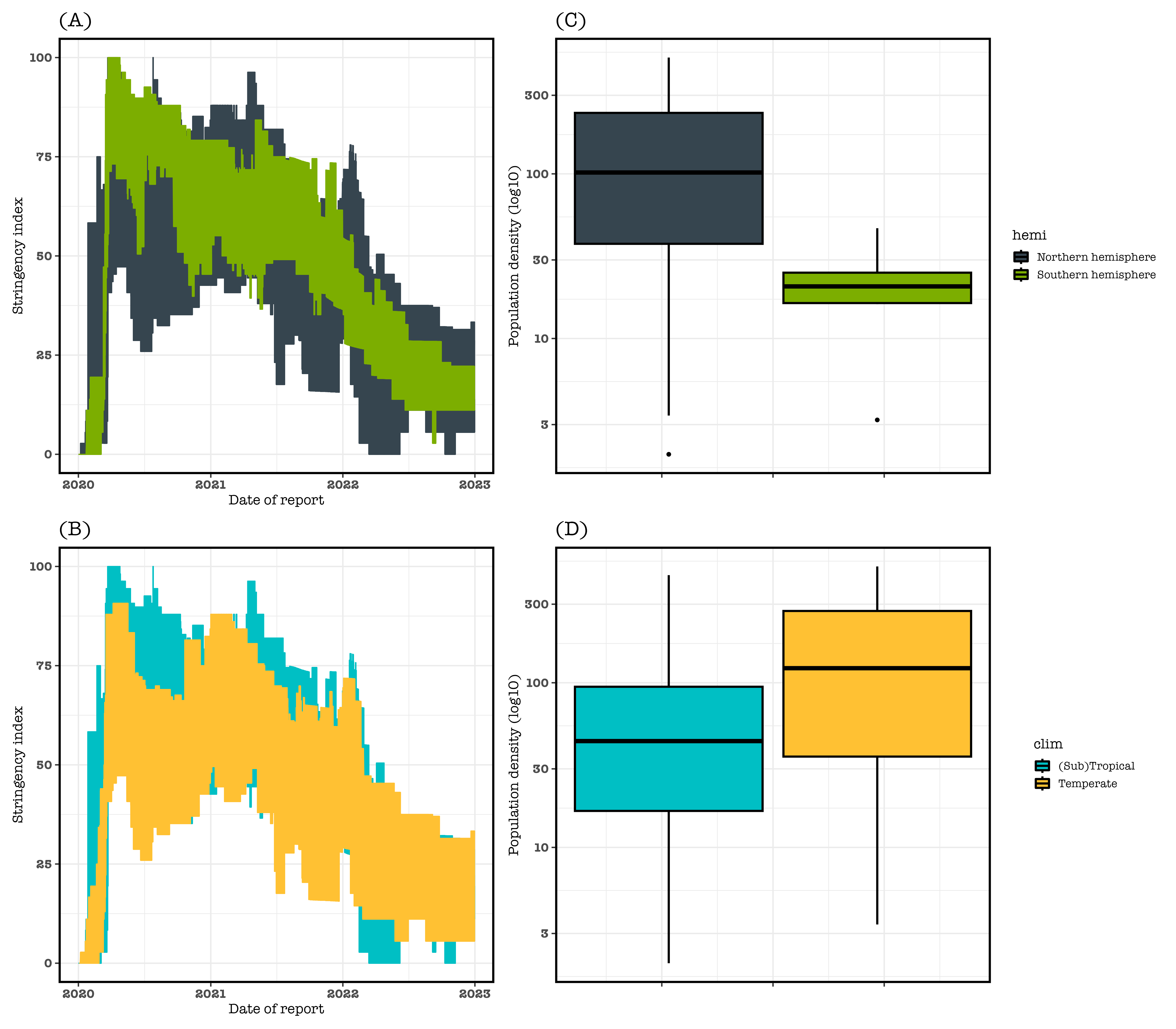 |
| --- |

Supplementary Figure 5. Distribution of contact stringency index and population density by hemisphere and climate zone. (A, B) The contact stringency index is sourced from the Oxford COVID-19 Government Response Tracker data and uses nine metrics to calculate the Government Stringency Index including school closures, workplace closures, cancellation of public events, restrictions on public gatherings, closures of public transport, stay-at-home requirements, public information campaigns, restrictions on internal movements, and international travel controls. The stringency index is stratified by hemisphere and climate zone, with value of 0 referring to no restriction and 100 to maximum restrictions. (C, D) Box plot showing the spread of individuals per square kilometer (population density), in each hemisphere and climate zone.


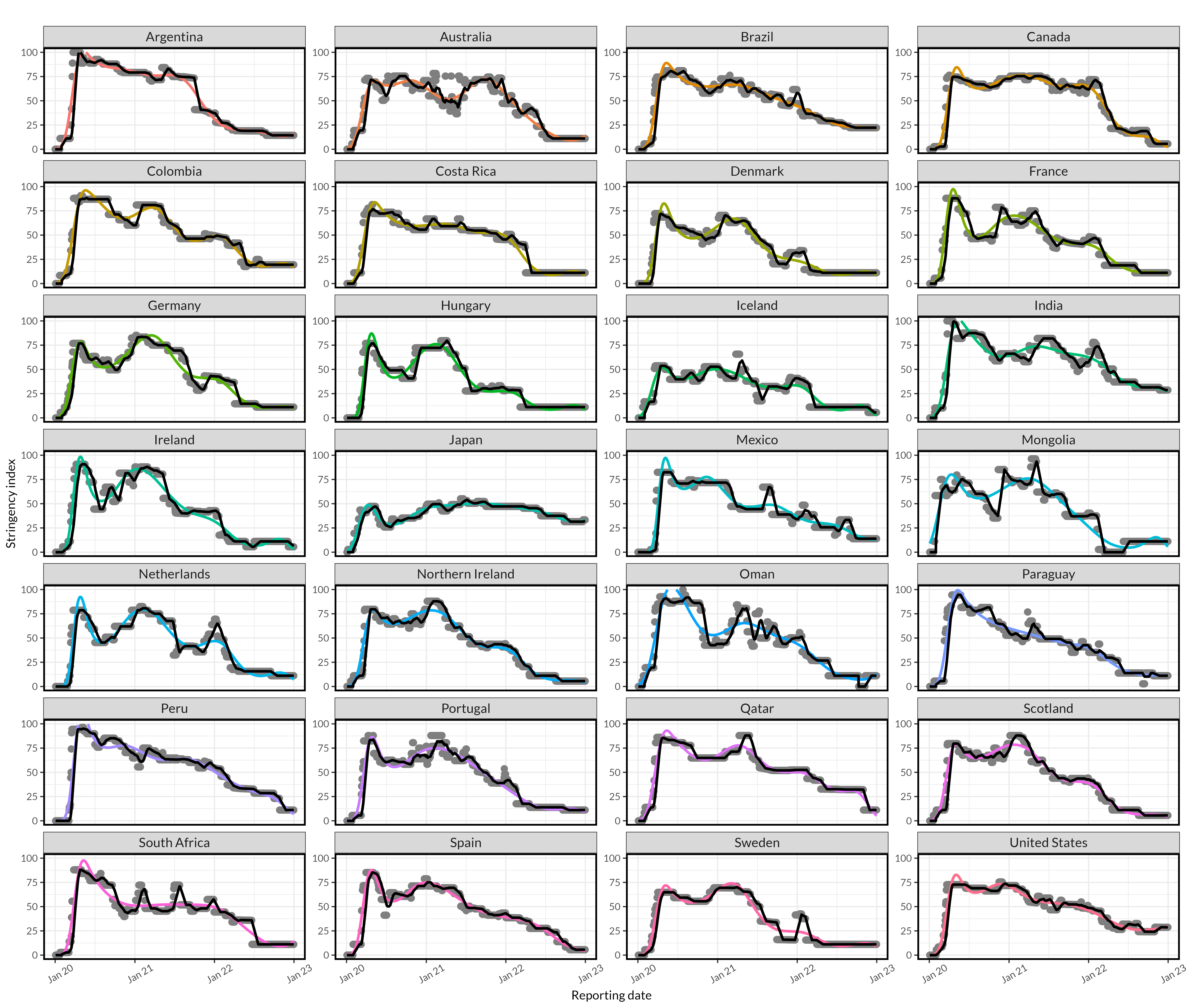


Supplementary Figure 6. Distribution of contact stringency index in 28 countries, globally. Thirty-days moving average (Black line), and generalised additive model (Color lines) fitted to reported stringency index (Gray points) to smooth out short-term effects of contact stringency. The contact stringency index is sourced from the Oxford COVID-19 Government Response Tracker data and uses nine metrics to calculate the Government Stringency Index including school closures, workplace closures, cancellation of public events, restrictions on public gatherings, closures of public transport, stay-at-home requirements, public information campaigns, restrictions on internal movements, and international travel controls. The stringency index has value of 0 referring to no restriction and 100 to maximum restrictions.

| 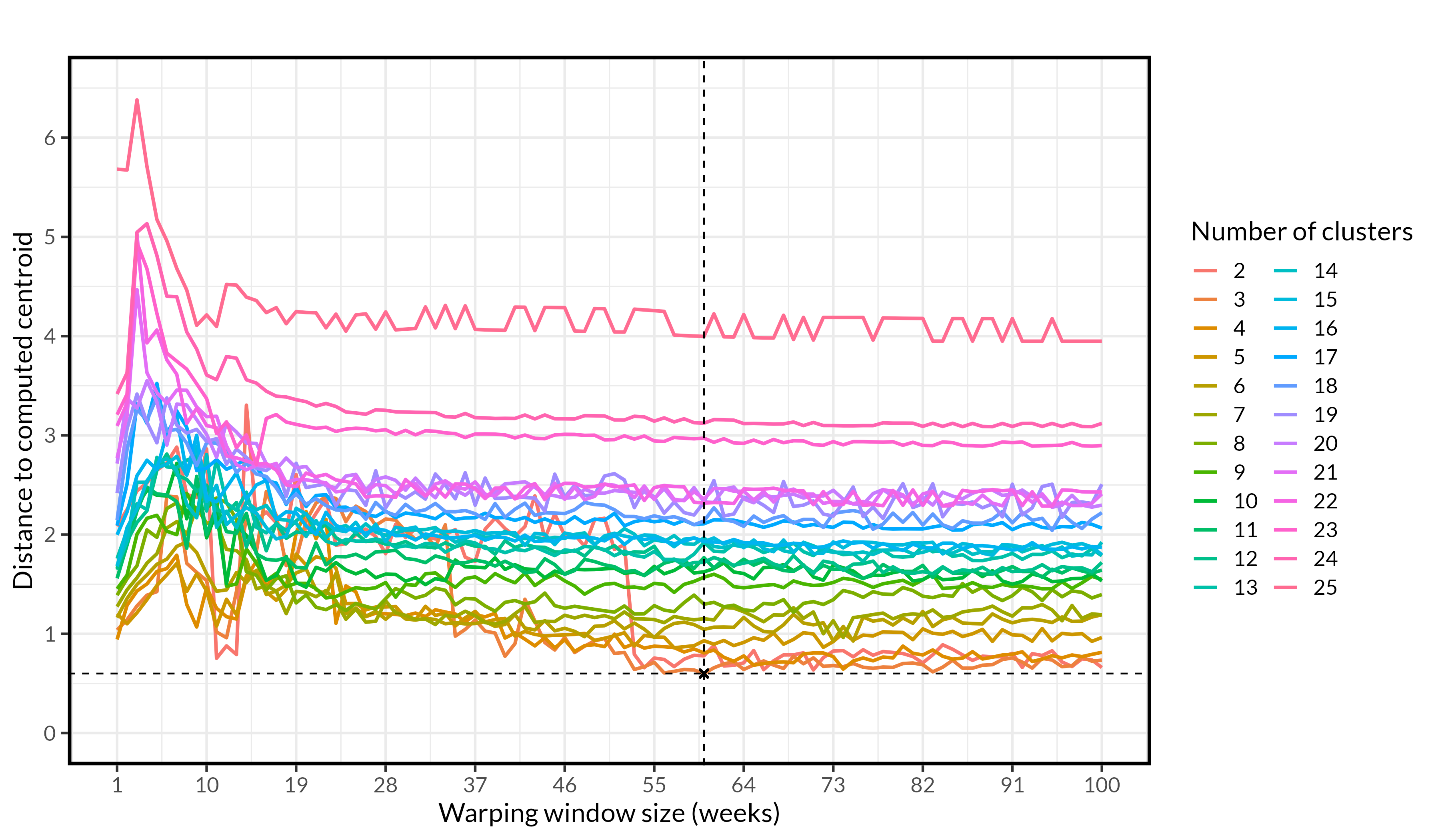 |
| --- |

Supplementary Figure 7. Evaluation of all possible number of clusters (between 2 and 25) and optimal DTW window size required to compute hierarchical clustering using Modified-Davies-Bouldin Cluster Validation Index (DB-CVI), which is based on calculating the distances from time series to their centroid, where the optimal DTW window size is the number of weeks at which distance is minimizes. The centroid of each cluster is computed using DTW barycentre averaging function. The optimal number of clusters is 3 with a corresponding DTW window size of *w*=60 (the black star where the vertical and horizontal dotted lines intersect). The next optimal number of clusters is 2 with corresponding DTW window size of *w=*71, and then 4 with corresponding DTW window size of *w=*74.

| 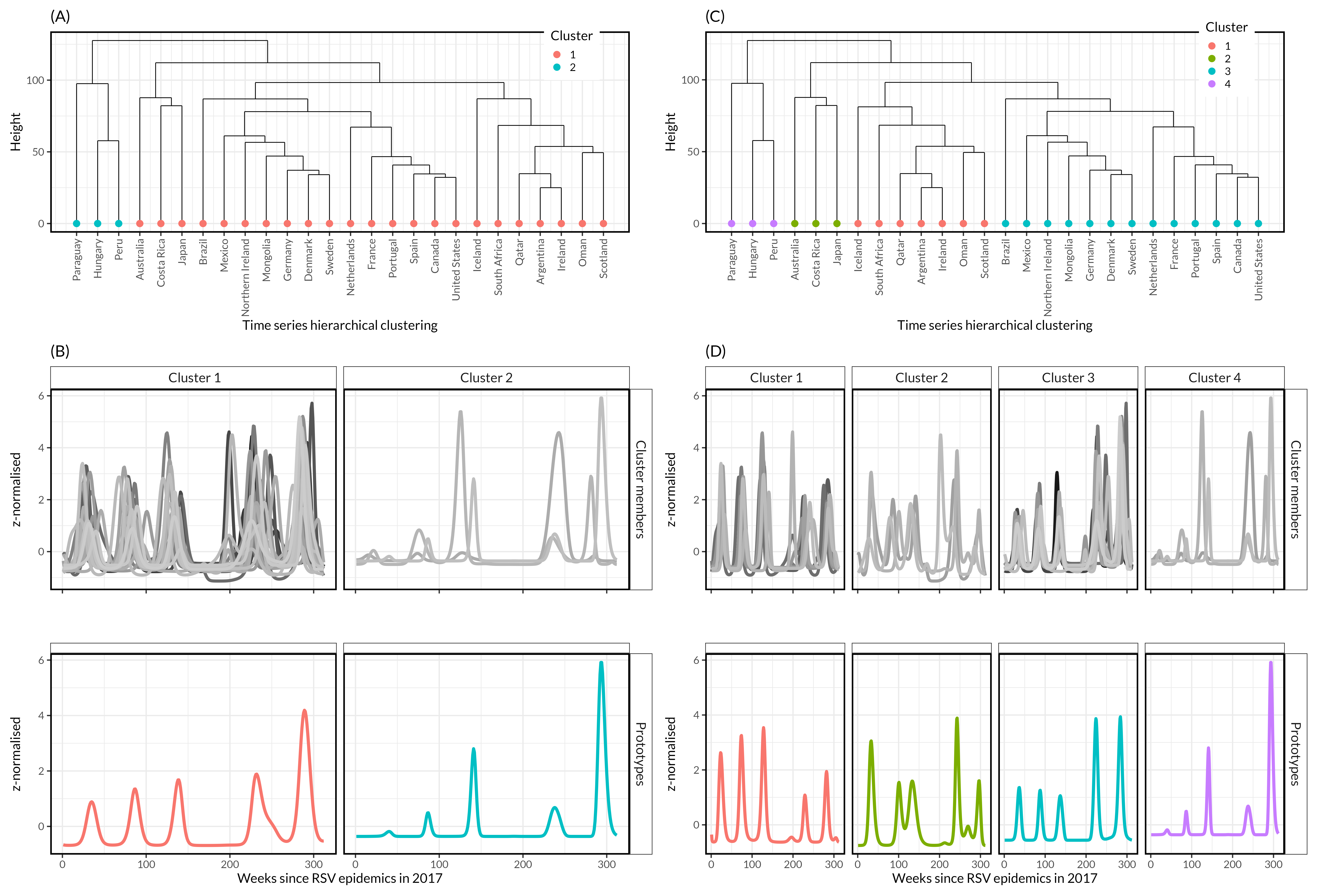 |
| --- |

Supplementary Figure 8. Sensitivity plots of time series dynamic time warping and time series classification using hierarchical clustering. (A, B) Dendrogram representing hierarchical clustering and its corresponding series prototypes if the number of clusters is set to 2 and warping window size to 71 as the optimal combination of parameters based on Modified Davies-Bouldin (DB) internal cluster validity index (CVI). (C, D) Dendrogram representing hierarchical clustering and its corresponding series prototypes if the number of clusters is set to 4 and warping window size to 74 based on DB and CVI.


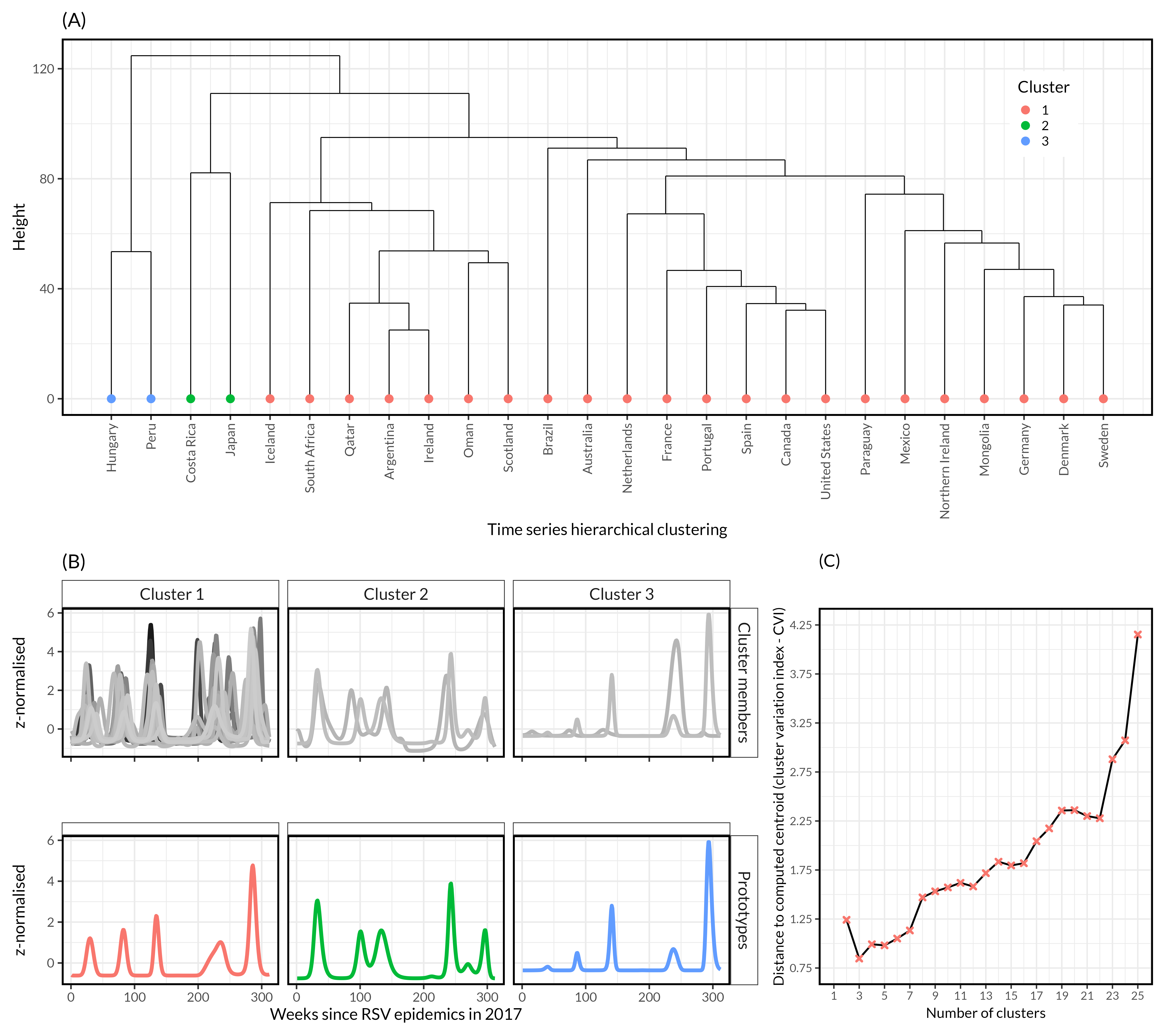


Supplementary Figure 9. Sensitivity plots of time series dynamic time warping and time series classification using hierarchical clustering. (A, B) Dendrogram representing hierarchical clustering and its corresponding series prototypes if the optimal number of clusters is set to 3 based on Modified Davies-Bouldin (DB) internal cluster validity index (CVI). (C) Evaluation of the possible number of clusters (between 2 and 25) using the entire warping window in order to compute hierarchical clustering using DB CVI which is based on calculating the distances from each P-spline time series to their centroid, where the optimal number of clusters is that which minimizes the distances. The centroid of each cluster is computed using DTW barycentre averaging function.

| 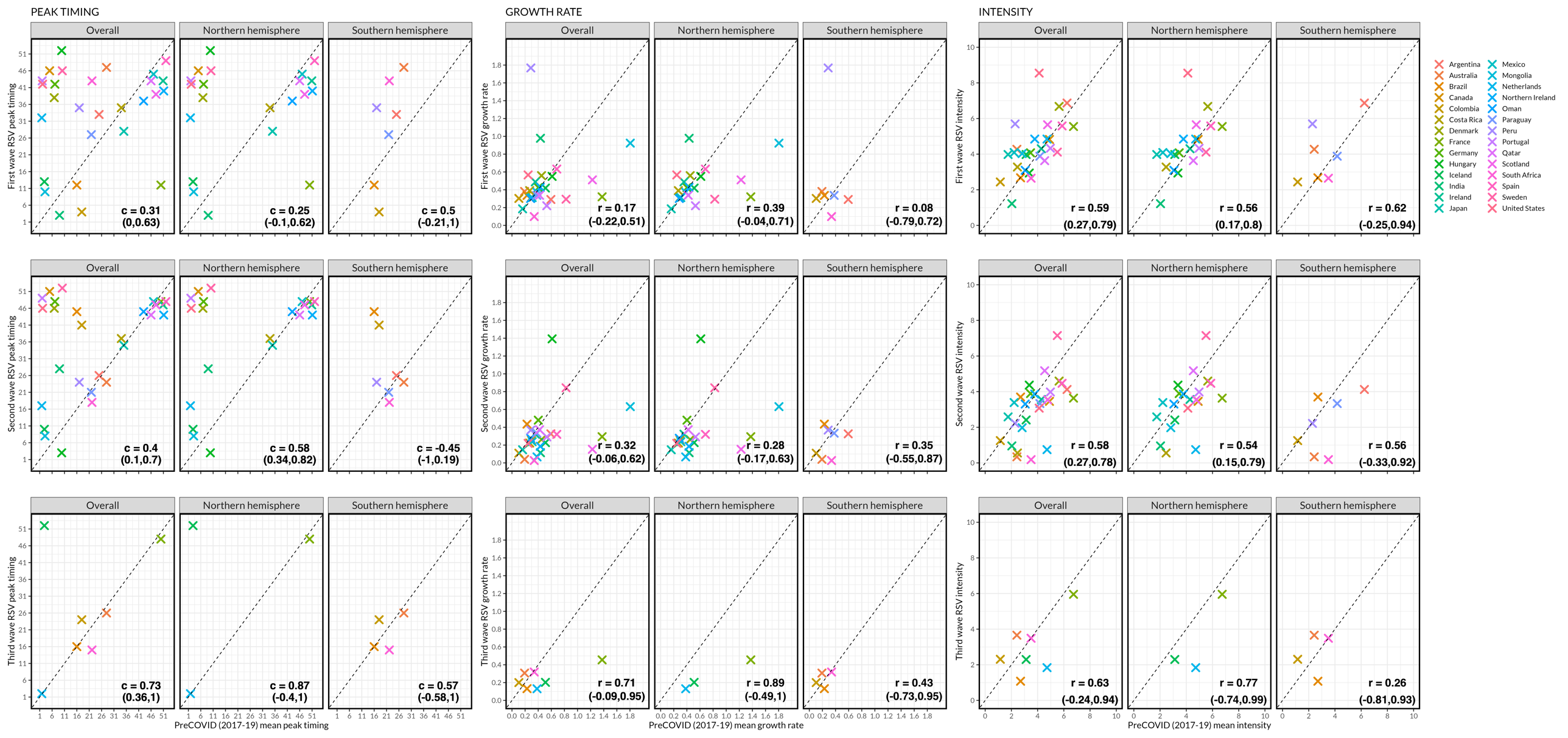 |
| --- |

Supplementary Figure 10. Respiratory syncytial virus (RSV) epidemic peak timing, growth rate and intensity in 28 countries. Comparing the RSV epidemic peak timing, growth rate and intensity between pre COVID-19 vs first, second and third waves of RSV in all countries, and by Northern and Southern hemispheres. The $c$ metric refers to the circular correlation coefficient whereas *r* metric is the Pearson’s correlation coefficient. The values in parentheses correspond to the 95% confidence intervals.

| 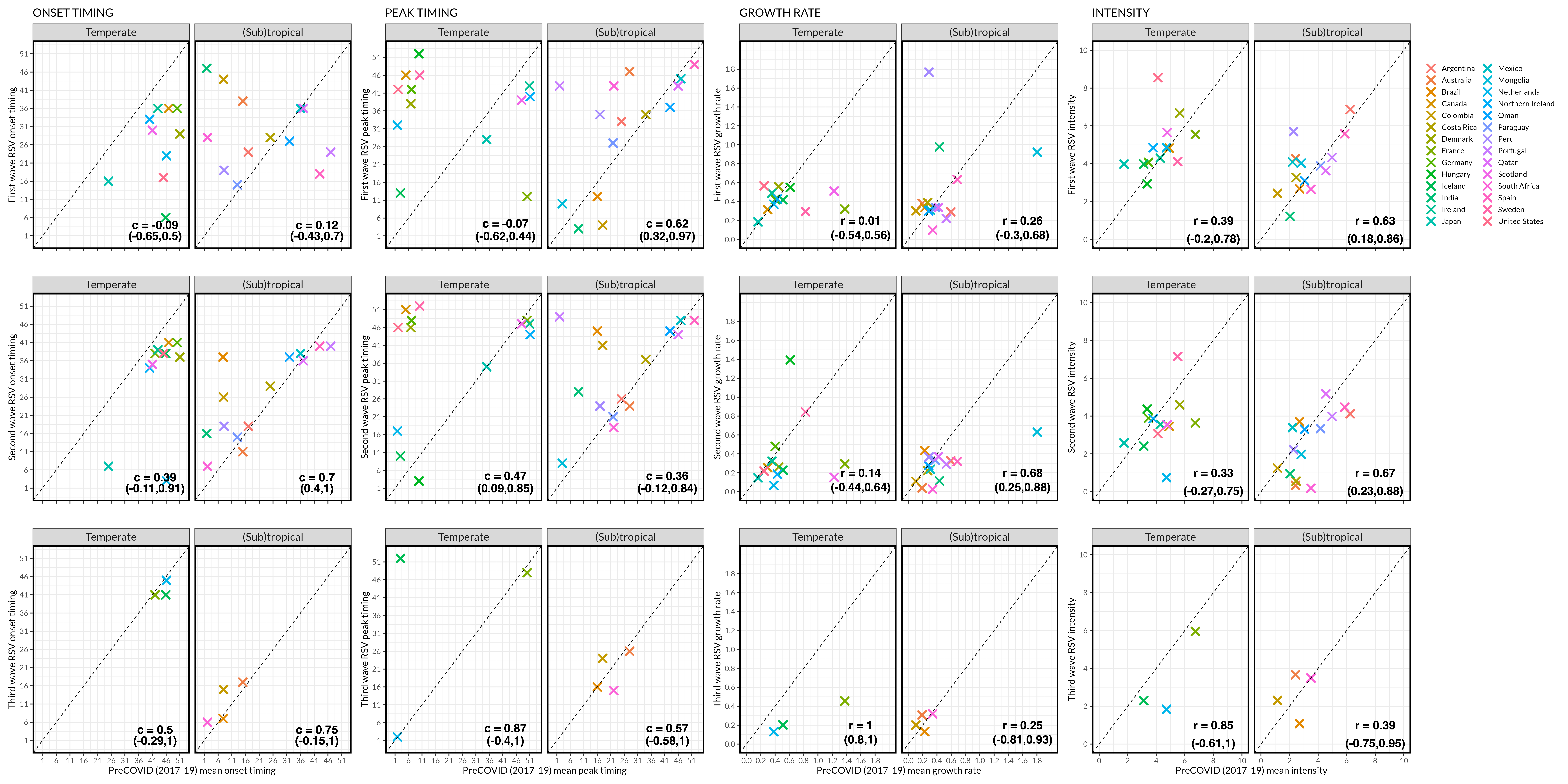 |
| --- |

Supplementary Figure 11. Respiratory syncytial virus (RSV) epidemic onset timing, peak timing, growth rate and intensity in 28 countries. Comparing the RSV epidemic onset timing, peak timing, growth rate and intensity between pre COVID-19 vs first, second and third waves of RSV in all countries, and by Temperate and (Sub)tropical. The $c$ metric refers to the circular correlation coefficient whereas *r* metric is the Pearson’s correlation coefficient. The values in parentheses correspond to the 95% confidence intervals.

| Supplementary Table 1. Correlation coefficients between different RSV waves post-COVID-19 and pre-COVID-19 mean values among countries that had a third wave of RSV. | | | |
| --- | --- | --- | --- |
| Season epidemic metric | Correlation between pre-COVID-19 and first wave of RSV | Correlation between pre-COVID-19 and second wave of RSV | Correlation between pre-COVID-19 and third wave of RSV |
| Onset† | -0.79 (-0.29, 0.48) | 0.38 (0.44, 0.95) | 0.97 (0.88, 1.00) |
| Peak† | 0.24 (0, 0.63) | -0.31 (-0.1, 0.70) | 0.73 (0.36, 1.00) |
| Growth rate‡ | 0.05 (-0.73, 0.78) | 0.35 (-0.55, 0.87) | 0.71 (-0.09, 0.95) |
| Intensity‡ | 0.80 (0.11, 0.97) | 0.36 (-0.54, 0.87) | 0.63 (-0.24, 0.94) |
| † Estimated with circular correlation coefficient  ‡ Estimated with Pearson’s correlation coefficient  Countries that had 3 waves of RSV following COVID-19 pandemic included Australia, Brazil, Colombia, France, Iceland, Netherlands, South Africa  The values in parentheses correspond to the 95% confidence intervals | | | |

| 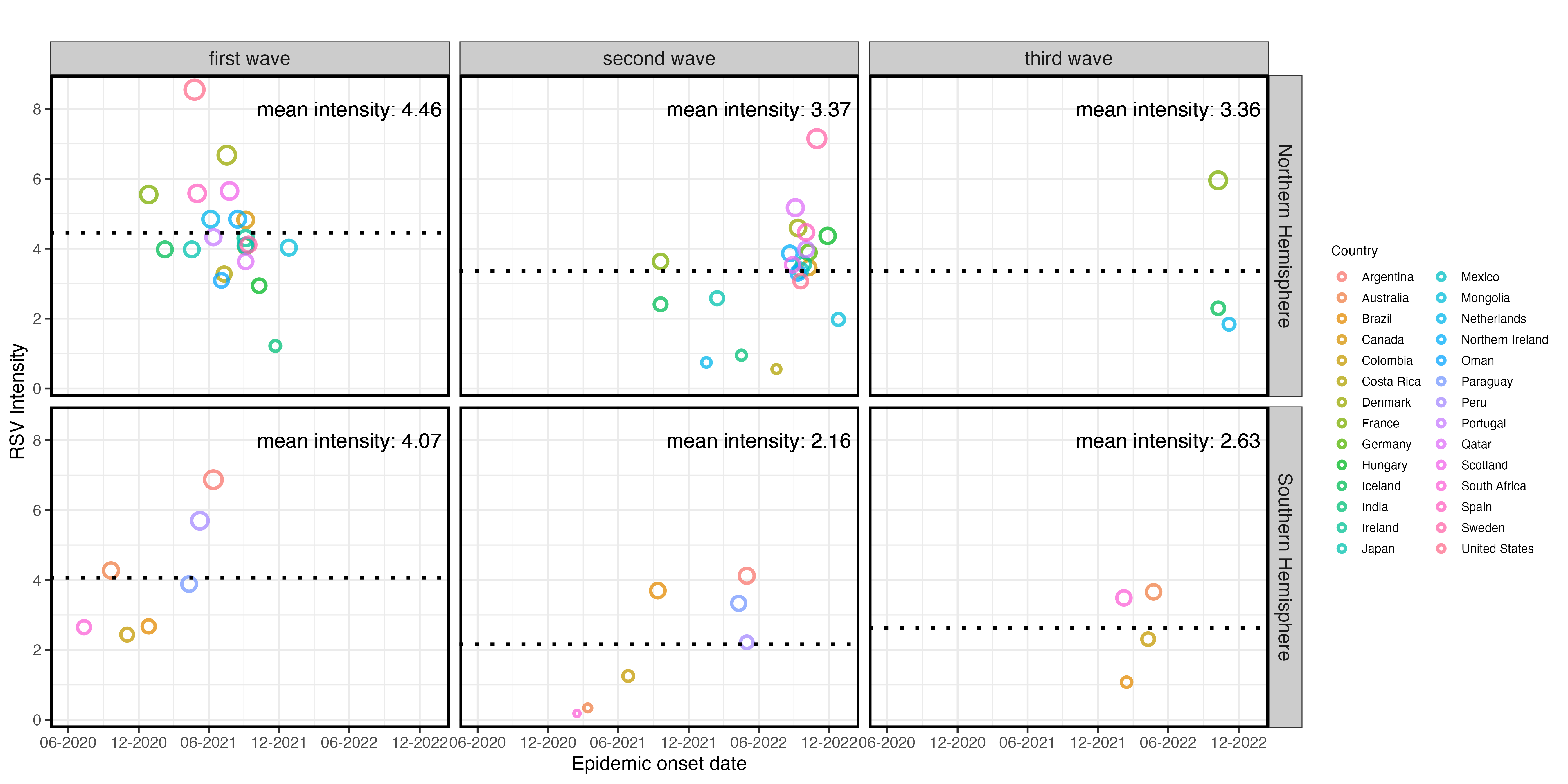 |
| --- |

Supplementary Figure 12. The relationship between respiratory syncytial virus (RSV) epidemic onset timing and intensity during the first, second and third waves. This plot shows relatively high mean RSV intensity in the Northern and Southern hemispheres during the first wave of RSV following COVID-19 pandemic, followed by relatively low intensity in the Northern and Southern hemispheres during the second and third waves. The black dashed lines represent the mean intensity.

| 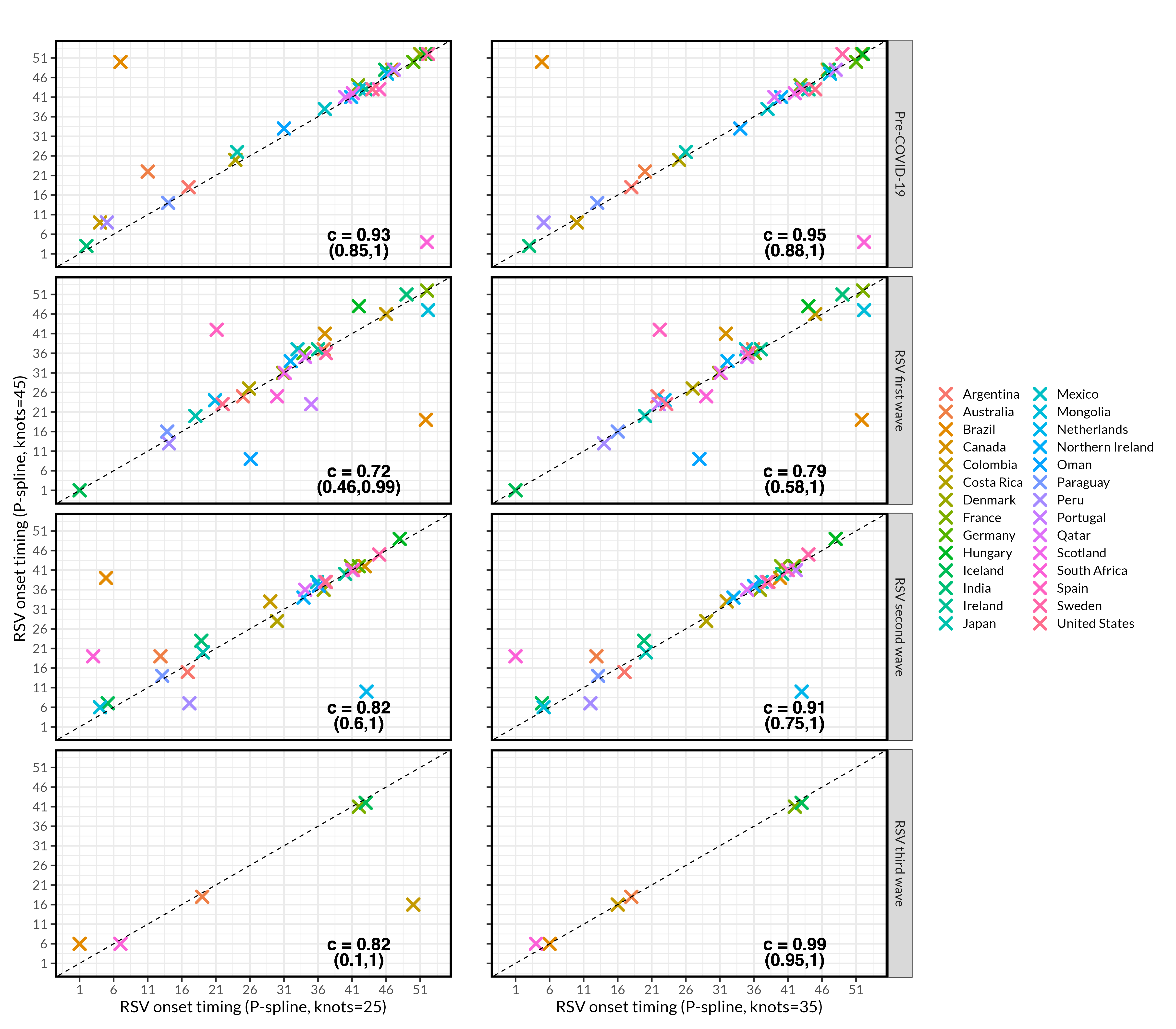 |
| --- |

Supplementary Figure 13. Sensitivity analysis of fitting different GAM P-spline models with varying number of knots on respiratory syncytial virus (RSV) epidemic onset timing. This plot shows very high correlation between RSV onset timing estimates obtained from fitting P-splines with 45 knots vs a base scenario of 25 knots, and alternative scenario of 35 knots across all epidemic periods (pre-COVID-19 pandemic, post-COVID-19 pandemic first wave, second wave, and third wave). The values in parentheses correspond to the 95% confidence intervals. These results signify that P-spline model fits were less sensitive to the choice of the number of knots between 25, 35 or 45.

Reference

1. Aghabozorgi S, Seyed Shirkhorshidi A, Ying Wah T. Time-series clustering – A decade review. Information Systems. 2015;53:16–38.

2. Giorgino T. Computing and Visualizing Dynamic Time Warping Alignments in R: The dtw Package. Journal of Statistical Software. 2009;31:1–24.

3. Sardá-Espinosa A. Time-Series Clustering in R Using the dtwclust Package. The R Journal. 2019;11:22.

4. Meyer D, Buchta C. proxy: Distance and Similarity Measures. R package. 2019.

5. Sakoe H, Chiba S. Dynamic programming algorithm optimization for spoken word recognition. IEEE Transactions on Acoustics, Speech, and Signal Processing. 1978;26:43–9.

6. Ratanamahatana C, Keogh EJ. Everything you know about Dynamic Time Warping is Wrong. 2004.

7. Dau HA, Silva DF, Petitjean F, Forestier G, Bagnall A, Mueen A, et al. Optimizing dynamic time warping’s window width for time series data mining applications. Data Min Knowl Disc. 2018;32:1074–120.

8. Hastie T, Tibshirani R, Friedman J. The Elements of Statistical Learning. New York, NY: Springer; 2009.

9. Hastie T, Tibshirani R. Generalized Additive Models. Statistical Science. 1986;1:297–310.

10. Artin B. pspline.inference: Estimation of Characteristics of Seasonal and Sporadic Infectious Disease Outbreaks Using Generalized Additive Modeling with Penalized Basis Splines. 2021.

11. Wood S. mgcv: Mixed GAM Computation Vehicle with Automatic Smoothness Estimation. 2023.

12. Eilers PHC, Marx BD. Flexible Smoothing with B-splines and Penalties. Statistical Science. 1996;11:89–102.

13. Zheng Z, Warren JL, Artin I, Pitzer VE, Weinberger DM. Relative timing of respiratory syncytial virus epidemics in summer 2021 across the United States was similar to a typical winter season. Influenza and Other Respiratory Viruses. 2022;16:617–20.

14. Boxtel G van, Tom S, Paul K, Abbott B, Aguado J, Annamalai M, et al. gsignal: Signal Processing. 2022.

15. Parag KV, Thompson RN, Donnelly CA. Are Epidemic Growth Rates More Informative than Reproduction Numbers? Journal of the Royal Statistical Society Series A: Statistics in Society. 2022;185 Supplement_1:S5–15.

16. Lund U, Agostinelli C, Arai H, Gagliardi A, García-Portugués E, Giunchi D, et al. circular: Circular Statistics. 2022.

17. Fisher RA. Frequency Distribution of the Values of the Correlation Coefficient in Samples from an Indefinitely Large Population. Biometrika. 1915;10:507–21.

18. Sauerbrei W, Perperoglou A, Schmid M, Abrahamowicz M, Becher H, Binder H, et al. State of the art in selection of variables and functional forms in multivariable analysis—outstanding issues. Diagnostic and Prognostic Research. 2020;4:3.
